# Accelerated marsh erosion following the *Deepwater Horizon* oil spill confirmed, ameliorated by planting

**DOI:** 10.1038/s41598-022-18102-1

**Published:** 2022-08-13

**Authors:** Scott Zengel, Zachary Nixon, Jennifer Weaver, Nicolle Rutherford, Brittany M. Bernik, Jacqueline Michel

**Affiliations:** 1 Research Planning, Inc. (RPI), 247 E. 7th Avenue, Suite 200, Tallahassee, FL 32303 USA; 2 Research Planning, Inc. (RPI), 1121 Park Street, Columbia, SC 29201 USA; 3 Office of Response and Restoration, Emergency Response Division, National Oceanic and Atmospheric Administration (NOAA), 7600 Sand Point Way NE, Seattle, WA 98115 USA; 4 Department of Ecology and Evolutionary Biology, Tulane University, New Orleans, LA 70118 USA; 5 Present Address: Gulf Coast Ecosystem Restoration Council, 500 Poydras Street, Suite 1117, New Orleans, LA 70130 USA

**Keywords:** Environmental sciences, Environmental impact, Ecology, Restoration ecology, Wetlands ecology

## Abstract

Multiple studies have examined the effects of the *Deepwater Horizon* oil spill on coastal marsh shoreline erosion. Most studies have concluded that the spill increased shoreline erosion (linear retreat) in oiled marshes by ~ 100–200% for at least 2–3 years. However, two studies have called much of this prior research into question, due to potential study design flaws and confounding factors, primarily tropical cyclone influences and differential wave exposure between oiled (impact) and unoiled (reference) sites. Here we confirm that marsh erosion in our field experiment was substantially increased (112–233%) for 2 years in heavily oiled marsh after the spill, likely due to vegetation impacts and reduced soil shear strength attributed to the spill, rather than the influences of hurricanes or wave exposure variation. We discuss how our findings reinforce prior studies, including a wider-scale remote sensing analysis with similar study approach. We also show differences in the degree of erosion among oil spill cleanup treatments. Most importantly, we show that marsh restoration planting can drastically reduce oiled marsh erosion, and that the positive influences of planting can extend beyond the immediate impact of the spill.

## Introduction

**Figure 1:**
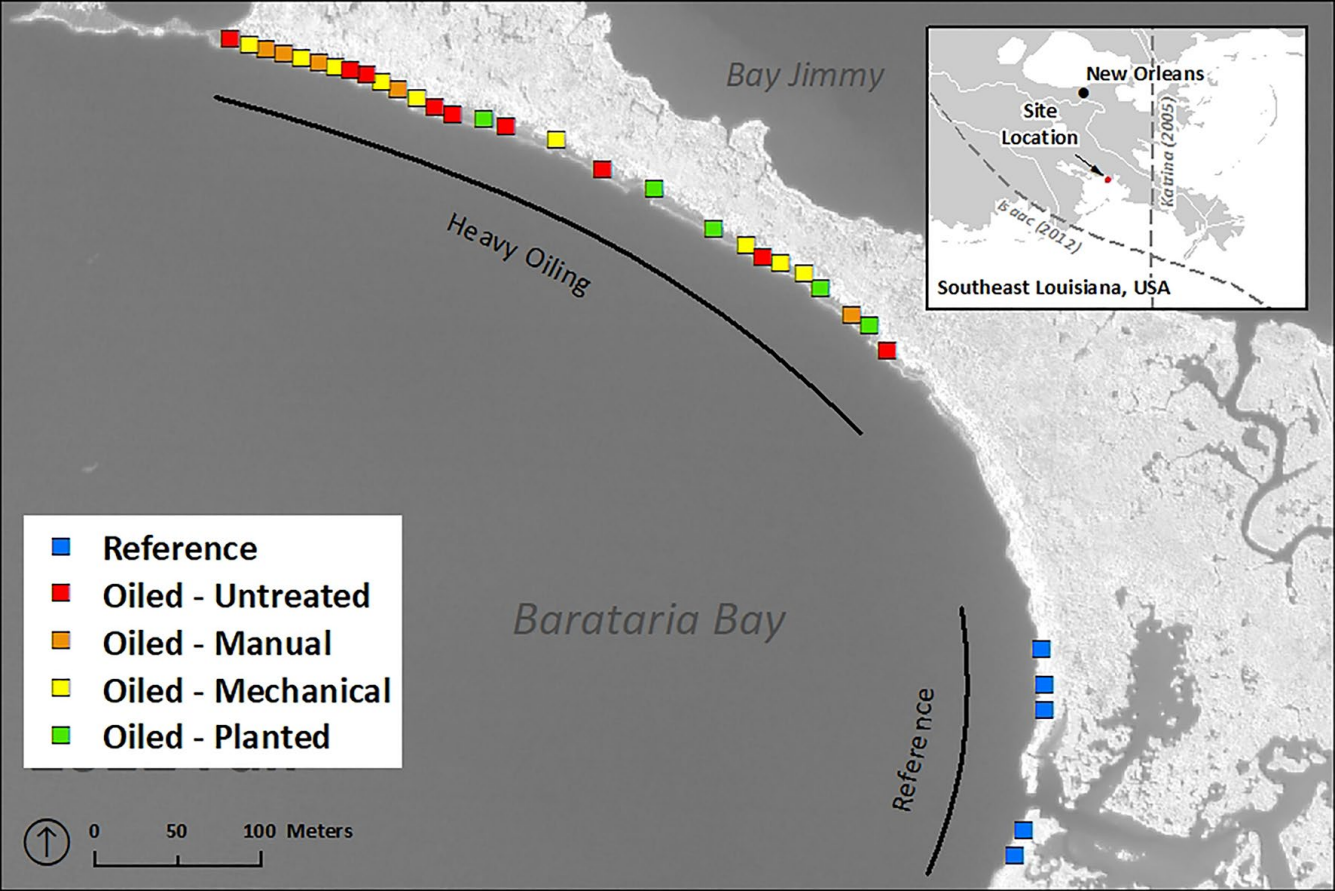
Field experiment map with study sites by marsh oiling/treatment class, including reference. The study area is salt marsh dominated by *Spartina alterniflora*, located on Barataria Bay, part of the Mississippi River Delta, Southeast Louisiana, USA. Inset map includes tracks for Hurricane Katrina (2005, east of study site) and Hurricane Isaac (2012, south and west of study site). Figure generated by the authors for this research using ArcGIS v10.8.1 (https://www.esri.com/en-us/arcgis/products/arcgis-desktop/overview).

Marine oil spills continue to be a worldwide concern, reinforced by periodic large incidents such as the *Deepwater Horizon* crude oil spill (2010) which affected ~ 1000 km of coastal wetland shorelines in the Mississippi River Delta, USA^1^. Coastal wetlands, such as salt marshes and mangroves, are of major conservation and restoration interest due to human-induced losses and corresponding reductions in ecosystem services such as storm protection, water quality improvement, fisheries production, and carbon sequestration^2,3^. Coastal marsh shoreline erosion in the Mississippi River Delta is particularly alarming due to high background rates of erosion linked with human-related causes, including relative sea level rise, lack of sediment input due to river modifications, and landscape alteration from oil and gas activities^2,4^. Accordingly, multiple studies have examined the added effects of the *Deepwater Horizon* oil spill on coastal marsh shoreline erosion, primarily in salt marshes dominated by *Spartina alterniflora* and to a lesser degree *Juncus roemerianus*^5–20^. Most studies concluded that the spill increased shoreline erosion (linear retreat) in oiled marshes by ~ 100–200% over 2–3 years, as a result of oil spill impacts to marsh vegetation and soils (Table 1). However, two studies, Deis et al.^18^ and Challenger et al.^19^, have called nearly all of this prior research into question, based on potential study design flaws and confounding factors, primarily tropical cyclone influences and differential wave exposure between oiled and unoiled or reference sites^18,19^. Here we report on the results of a field experiment (Fig. 1) consisting of 7 years of field data coupled with an overlapping 62-year record of digital aerial photography, to examine marsh shoreline erosion and the questions raised by these two papers. Our most interesting results are based on a Before–After-Control-Impact (or BACI) design^21,22^, whereas all preceding papers on this topic, including our own, have employed Control-Impact or Before–After approaches. BACI experimental designs are considered optimal for isolating environmental impacts from other sources of variability, including natural variation among impact and control sites, as well as variability over time. In addition to looking at the effect of oiling on marsh shoreline erosion, we also report on the potential longer-term influences of oil spill cleanup treatments and subsequent restoration planting. These topics have been largely unaddressed in prior oiled marsh erosion studies.

**Table 1:** Summary of *Deepwater Horizon* oil spill (DWH) marsh erosion studies to date.

Marsh erosion study	DWH erosion effect^a^	DWH oiling levels with erosion effect	Cleanup treatment effect^b^	Planting treatment effect^b^	Time-period of study^c^	Duration of DWH erosion effect	Study methods (primary design)^d^	Geographic scale^e^
Silliman et al.^5^	Yes (125%)	Heavy	–	–	2010–2012	1.5 years	Field (CI)	Small
McClenachan et al.^6^	Yes (hypothesized)	Moderate to heavy	–	–	2010–2012	2 years (hypothesized)	Field (CI)	Small, localized
Zengel et al.^7^	Yes (133–250%)	Heavy (very heavy)	Yes (−) (partly, indirect)	Yes (+) (direct)	2010–2012	2 years	Field (CI)	Small, localized
Gibeaut et al.^8^	Yes (44%)	Heavy (treated)	Undetermined	–	2010–2013	2.6 years	Remote sensing (CI)	Large (BB)
Lin et al.^9^	Yes (elev. loss)	Heavy	–	–	2011–2013	1.5 years	Field (CI)	Small
Beland et al.^10^	Yes (100%, area)	Heavy	–	–	2010–2012	2.5 years	Remote sensing (CI)	Large (BB)
McClenachan ^11^	Yes (50%)	Moderate to heavy (direct), light to none (indirect)	–	–	2010–2015 (field), 1998–2013 (RS)	2 years (direct), 4–5 years (direct and indirect)	Field (CI), remote sensing (BA)	Small, localized
Turner et al.^12^	Yes (224%)	Oiled (light-heavy)	–	–	1989–2012	2.5 years	Remote sensing (CI)	Large (LA)
Rangoonwala et al.^13^	Yes	Light to heavy (mostly moderate to heavy)	–	–	2009–2012	2.5 years	Remote sensing (BA)	Large (BB)
Silliman et al.^14^	Yes (73–117%)	Heavy	–	–	2010–2013	2–3 years	Field (CI)	Large (LA-MS-AL)
Khanna et al.^15^	Yes (31–84%, area)	Heavy	Undetermined	–	2010–2012	1–2 years	Remote sensing (CI)	Large (BB)
Beland et al.^16^	Yes (52%, area)	Heavy	–	–	2006–2016	3 years	Remote sensing (BA)	Large (BB)
Powers et al.^17^	Yes (133%)	Moderate to heavy	No	–	2010–2013	3 years	Field (CI)	Large (LA-MS-AL)
Deis et al.^18^	No	–	–	–	1998–2013	–	Remote sensing (BA)	Small
Challenger et al.^19^	Yes (97–107%)	Heavy to very heavy (treated)	Undetermined	–	2010–2015	1 year (heavy), 3–4 years (very heavy, treated)	Field (CI)	Large (LA)
Bernik et al.^20^	–	Heavy (treated)	Undetermined	Yes (+) (direct)	2012–2013	–	Field (CI)	Small, localized
Zengel et al., present study	Yes (112–233%)	Heavy (very heavy)	Yes (−) (partly, direct)	Yes (+) (direct)	2010–2016 (field), 1956–2018 (RS)	2 years	Field (CI), remote sensing (BACI)	Small, localized

^a^Percent increase in linear erosion rate in parentheses unless otherwise noted.

^b^Positive (+) or negative (−) effects indicated, undetermined = studies that included treated marsh but without direct comparisons to untreated sites with similar oiling levels or with inconclusive results.

^c^*RS* remote sensing.

^d^*CI* control-impact, *BA* before–after, *BACI* before–after-control-impact.

^e^*BB* Barataria Bay (Louisiana), *LA* wider Louisiana, *MS* Mississippi, *AL* Alabama, all smaller-scale studies were in portions of Barataria Bay or associated bays (e.g., Bay Jimmy, Bay Batiste, Wilkinson Bay).

## Oiled marsh erosion and soil shear strength, field-based control-impact

We start by looking at our field erosion data, collected through 6 years post-spill (7 years total), to determine the duration of oiling effects, and to look for longer-term cleanup treatment influences. We previously observed increased erosion in our heavily oiled marsh sites for a 2-year period after the spill, the duration of our study at that time^7^. We also observed no major differences in erosion among oiled sites that were untreated versus those with manual cleanup treatments. In a separate but related experiment, using slightly different methods, there were indirect indications that mechanical cleanup treatments may have further worsened erosion and direct evidence that planting limited erosion in mechanically treated sites over a 1 year study period^7^.

In the present study, our field-based comparisons of oiling/treatment categories included reference, oiled and untreated, oiled and manually treated, and oiled and mechanically treated sites. Untreated sites had no active cleanup (i.e., natural recovery), an approach which is commonly prescribed for oiled marshes (see Zengel et al.^7^ for background on the trade-offs of typical oiled marsh treatment tactics). Manual cleanup treatment involved raking, cutting, and removal of oiled wrack, oiled vegetation mats (laid over oiled and dead vegetation that was still rooted), and underlying thick oil on the substrate by small crews using hand tools, to remove surface oiling to the extent possible and to better expose remaining oil to natural weathering and degradation processes^7^. Hand crews used walking boards to minimize foot traffic on the marsh surface. Mechanical cleanup treatment involved mechanized grappling to remove oiled wrack and mechanized raking, cutting, and scraping to remove or reduce oiled vegetation mats and oil on the substrate. The mechanical treatments were applied using long-reach hydraulic arms mounted on shallow-draft barges and large airboats stationed just seaward of the marsh shoreline^7,23^. Mechanical treatment was aimed toward the same goals as manual cleanup but with anticipated increases in speed and scale; however, mechanical treatment can also be less precise, resulting in removal of soils, mixing of oil into the substrate, etc. Oiling conditions were highly consistent across all the oiled sites, characterized as heavy oiling using Shoreline Cleanup Assessment Technique (SCAT) methods^7^, although we agree that oiling in our sites could be considered “very heavy” as proposed by others^19^. Oiling conditions consisted of a continuous 6–13 meters (m) wide oiling band along the marsh shoreline, with heavily oiled wrack and vegetation mats overlying a 2–3 centimeter (cm) layer of emulsified oil on the marsh surface with ~ 90–100% oil cover^7^. The heavily oiled sites all experienced complete or near complete vegetation die-off, with vegetation recovery spanning multiple years^7,23^. Nearby reference sites on the same shoreline had lighter to no oiling, intact vegetation structure, and no cleanup treatments^7^. Further details on oiling conditions, cleanup treatments, and vegetation response are included in our prior papers (including photographs)^7,23^.

**Figure 2:**
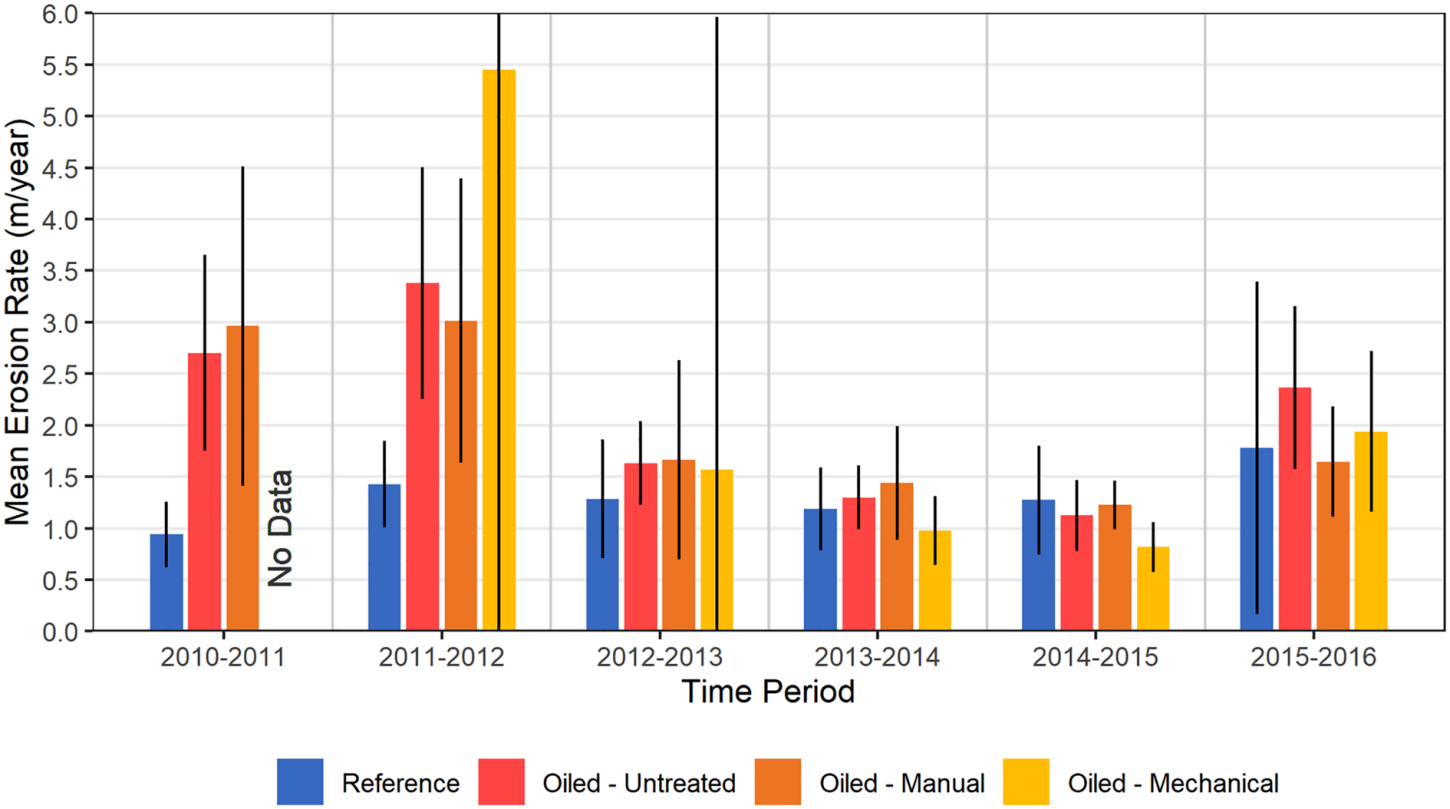
Field measured marsh shoreline erosion rates 2010–2016 (m yr^−1^). Data are means with 90% confidence intervals, n = 5 for Reference, 9 for Oiled-Untreated, 5 for Oiled-Manual, and 2–6 for Oiled-Mechanical treatments (n = 14–20 for Oiled sites combined) depending on year. Due to missing values, the desire to use as much data as possible, and the lack of clear differences among cleanup treatments, we pooled the oiled site data for statistical analysis. Marsh erosion rates differed among Reference and Oiled sites (F_1,17_ = 9.751, *p* = 0.006); among years (F_2.32,39.40_ = 2.703, *p* = 0.072); and for the interaction of oiling and year (F_2.32,39.40_ = 2.648, *p* = 0.076). Pairwise differences (Tukey’s test) among Reference and Oiled sites were observed for 2010–2011 (*p* = 0.003) and 2011–2012 (*p* = 0.001), but not for other years. See Supplementary Table S1 for detailed two-way mixed ANOVA results.

Annual shoreline erosion rates were determined each year by ground surveys using tape measures and differentially corrected GPS (± 10 cm horizontal accuracy) to measure shoreline position along established transects. In the present study, we observed 147–198% greater marsh shoreline erosion for the oiled versus reference sites over 2 years (2010–2011 and 2011–2012), with no clear differences among oiled sites with or without cleanup treatments (Fig. 2, Supplementary Table S1). There was some indication that mechanical treatment may possibly have worsened erosion in some sites in 2011–2012, which matched our field observations and prior experiments^7^, though our sample size was small and highly variable in this case.

**Figure 3:**
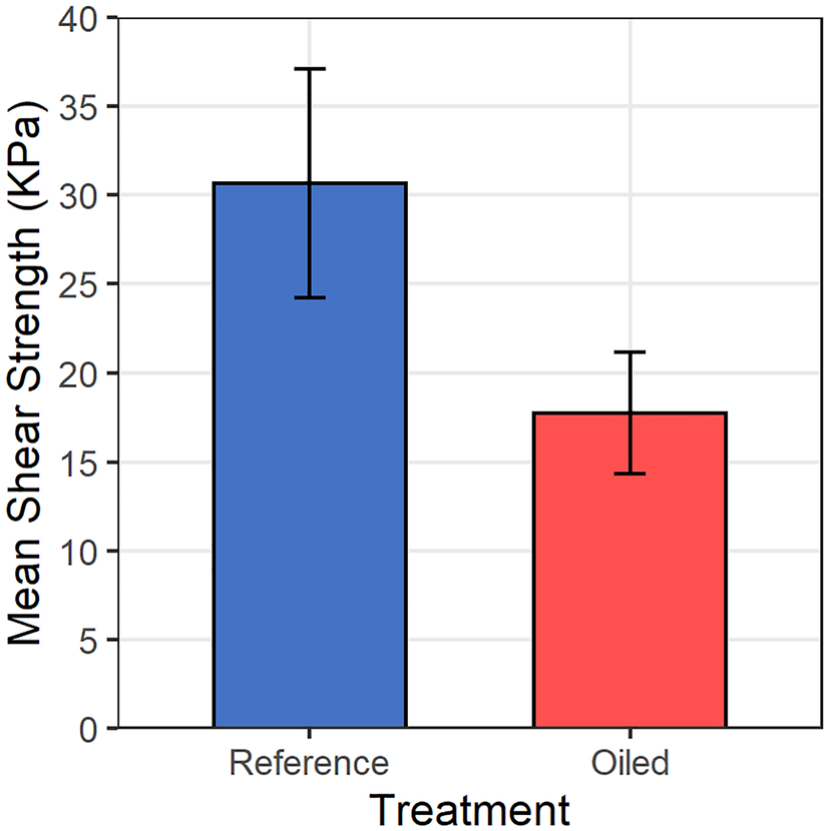
Field measured marsh soil shear strength 2011–2012 (KPa). Data are means with 90% confidence intervals, n = 4 for Reference and 13 for Oiled sites. Soil shear strength differences were observed among the Reference and Oiled sites (t_6.29_ = − 3.877, *p* = 0.007, Welch’s t-test). See Supplementary Table S2 for further details.

Potential causes for increased shoreline erosion in oiled marshes may lie most directly with the die-off of marsh vegetation, which baffles wave energy and binds marsh soils. Die-off of vegetation at the marsh edge was likely caused by several related factors, including thick persistent oiling covering all or most of the aboveground vegetation and soil surface, repetitive oiling, and penetration and mixing of oil into the soils, resulting in fouling and smothering effects on the plants such as interference with photosynthesis, gas exchange, thermal regulation, etc., leading to plant death. Our prior work showed substantial reductions (77–100%) in aboveground total plant cover (all species) and *Spartina alterniflora* cover (the dominant marsh species) in our oiled sites versus reference over 2010–2011 (and 45–99% reductions over 2011–2012)^7^. Belowground plant biomass was likely also reduced, although this was not measured in our study. However, belowground biomass was reduced in similarly oiled sites in studies by others^5,9,14,24^. Significant relationships have been established between marsh belowground biomass and soil shear strength (a measure of erosion potential)^25^, including in oiled marshes^9^, and belowground biomass is the main vegetation trait that resists erosion in coastal marshes^26^. Working in collaboration with us, Lin et al.^9^ conducted ancillary sampling in a subset of our study sites over the 2011–2012 period and provided their unpublished soil shear strength data (0–6 cm) for our use (their sampling did not include our planted sites). There was a 42% reduction in soil shear strength in the oiled sites relative to the reference sites (Fig. 3, Supplementary Table S2). Our reference site mean values are very similar to marsh soil shear strength values reported by others in our study region^9,27^, whereas our oiled site values are much lower. Thus, there is evidence that oiling affected both the marsh vegetation and the erodibility of the marsh soils, which likely led to the observed differences in marsh erosion between the reference and oiled sites. Oyster beds were not present near the marsh edge in our study area; thus, oyster cover impacts did not contribute to observed erosion differences between our oiled and reference sites (see Powers et al.^17^).

## Oiled marsh erosion reconsidered, remote sensing before–after-control-impact

Criticisms^18,19^ of prior *Deepwater Horizon* oiled marsh erosion papers, including ours^7^, would apply to the results above as well. The most interesting of these criticisms was that Hurricane Katrina (2005) increased baseline erosion rates in the region, and that this signal was mistaken for oil spill effects^18^. This criticism could apply if prior studies applied a potentially misleading baseline while either: (a) comparing pre-spill erosion rates among oiled and reference sites to justify a Control-Impact approach; or (b) comparing erosion rates in oiled sites pre- and post-spill in a Before–After design. In both cases, this concern may have potentially applied to studies using a longer-term historical baseline (as in our prior paper^7^) but could also apply to studies using a shorter baseline, such as 1 year^18,19^.

To explore the above idea in our own study, we extracted shorelines from a time-series of digital aerial photographs and used the U.S. Geological Survey’s Digital Shoreline Analysis System^28^ to model annual erosion rates for our field sites over the following time-periods: pre-Katrina/pre-spill (1956–2004), Hurricane Katrina (2004–2005), post-Katrina/pre-spill (2005–2010), spill impact (2010–2012), and post-spill (2012–2018) (see Supplementary Methods Table 1 for details on the aerial imagery used in our analysis). Our time-periods were similar but slightly different than those used by Deis et al.^18^ in that we used a longer “historical” pre-Katrina/pre-spill baseline, a shorter spill impact period (2 years vs. 3 years, based on our field data above and similar annual patterns in the remote sensing data over 2010–2016), and included longer term post-spill data. Our post-Katrina baseline period matched closely with Deis et al.^18^. In addition, with the remote sensing data, we were able to include comparable erosion data for the different cleanup treatments as well as for the planting sites (which were similarly oiled, mechanically treated, then hand planted in 2011 with transplanted local wild *Spartina alterniflora* bare root stems at a density of ~ 2–3 stems m^−2^ ^7,20^).

**Figure 4:**
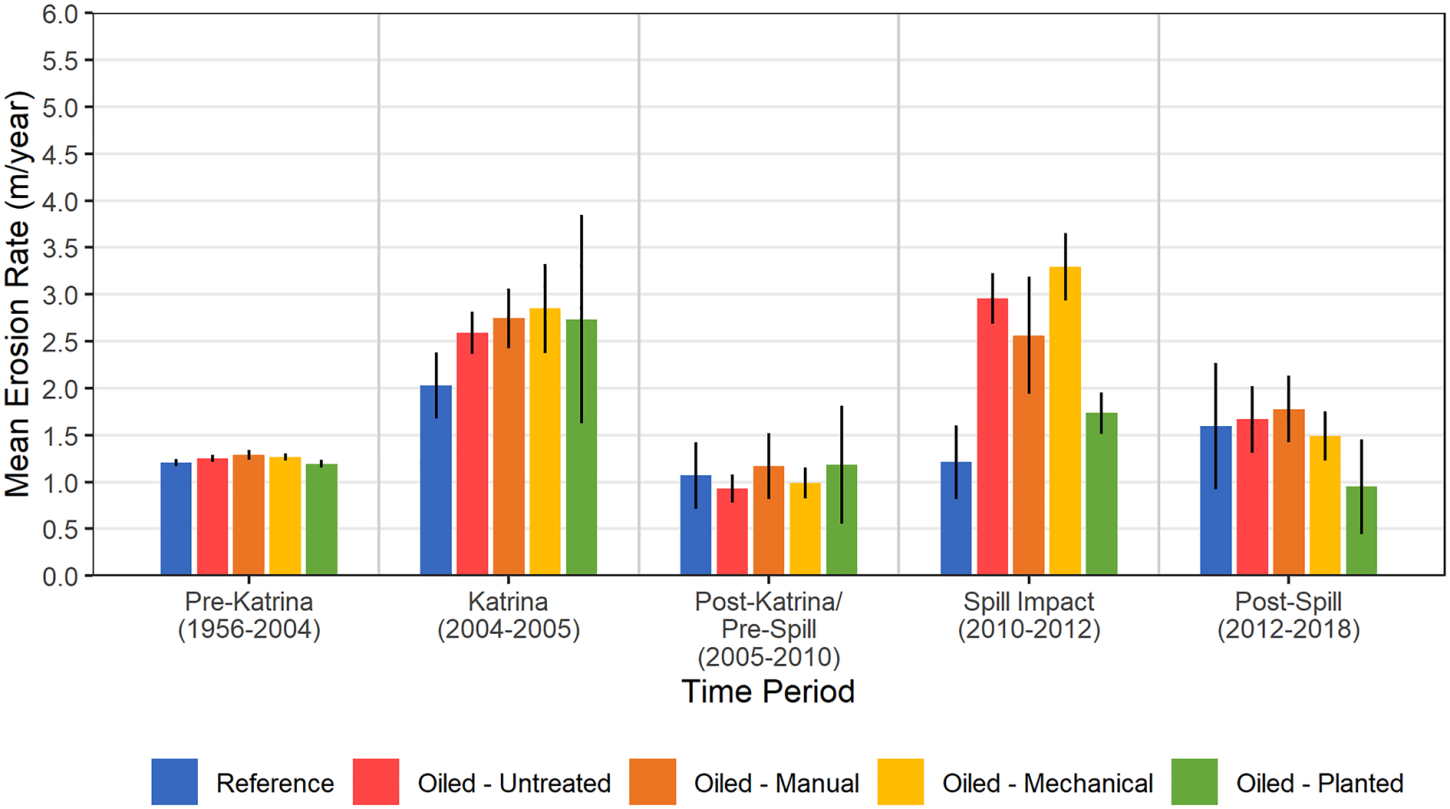
Remote sensing measured marsh shoreline erosion rates 1956–2018 (m yr^−1^). Data are means with 90% confidence intervals, n = 5 for Reference, 9 for Oiled-Untreated, 5 for Oiled-Manual, 9 for Oiled-Mechanical, and 5 for Oiled-Mechanical-Planted treatments. Marsh erosion rates differed among oiling/treatment categories (F_4,28_ = 9.368, *p* = 0.000); among time-periods (F_2.48,69.47_ = 62.210, *p* = 0.000); and for the interaction of oiling/treatment and time-period (F_9.92,69.47_ = 4.593, *p* = 0.000). In the post-Katrina/pre-spill period (2005–2010) erosion differences were not observed among any oiling/treatment classes, including Reference (*p* = 0.868 to 1.000). In the spill impact period (2010–2012) erosion differences were observed between: Reference versus all oiled classes (*p* = 0.000 to 0.001) except Planted (*p* = 0.410); between the Manual and Mechanical treatments (*p* = 0.063); and between Planted versus all other oiled classes (*p* = 0.000 to 0.065). In the post-spill period (2012–2018) erosion differences were observed between: Planted versus Untreated and Manual treatments (*p* = 0.066 and 0.072). Between the post-Katrina/pre-spill and spill impact time-periods, erosion differences were not observed within the Reference (*p* = 0.989) or Planted (*p* = 0.340) classes; however, differences were observed between these time-periods within each of the other oiled classes (*p* = 0.000 in all cases). See Supplementary Table S3 for detailed two-way mixed ANOVA results. Tukey’s test was used for all pairwise comparisons after ANOVA.

In our analysis, we observed 112–173% greater marsh shoreline erosion for the oiled and unplanted sites versus the reference sites in the spill impact period; whereas erosion in the reference and planted sites did not differ (Fig. 4, Supplementary Table S3). We also observed 47–90% greater erosion in the oiled and unplanted sites versus the oiled and planted sites (i.e., much less erosion was observed in the planted sites, even though they were not planted until 2011). There was also 29% greater erosion in the mechanical versus the manual treatment sites within the spill impact period, a stronger indication of cleanup treatment differences than in the field data, perhaps due to greater sample size. In contrast to the spill impact period, erosion differences were not observed among any oiling/treatment classes, including reference, in the post-Katrina/pre-spill baseline period (2005–2010), nor in any other time-periods, pre- or post-spill, with one important exception, the planted sites had less erosion than two of the three oiled and unplanted classes in the post-spill period (2012–2018) (Fig. 4, Supplementary Table S3). Differences in erosion were not observed between the post- Katrina/pre-spill (2005–2010) and spill impact (2010–2012) time-periods (the main before–after comparison) within either the reference or planted sites; whereas erosion increases of similar magnitude to our oiled versus reference comparisons were observed between these time periods within each of the oiled classes that were not planted. For instance, erosion in the oiled and mechanical treatment sites without planting increased by 233% between the post-Katrina/pre-spill period and the spill impact period (Fig. 4, Supplementary Table S3). This example re-emphasizes the clear importance of planting in reducing substantial shoreline erosion.

Several *Deepwater Horizon* oil spill marsh erosion studies referenced Hurricane Isaac (2012) as another major influencer on marsh erosion^10,11,13,15,16,18,19^. These papers argued that oiling may have weakened the marsh, leading to even greater erosion during Hurricane Isaac, or that Hurricane Isaac alone may have resulted in erosion impacts that were incorrectly attributed to the oil spill^18,19^. The latter argument included references to our prior findings^7^. We addressed this question directly using digital aerial photographs taken several days before and after the passage of Hurricane Isaac. We re-ran our annual erosion models and the corresponding analyses, omitting the shoreline extracted from the post-Isaac photography at the end of the spill impact period (2010–2012). This removed approximately 1 week of data from the end of this period, but also removed any erosion influence caused by the hurricane. The results from this alternative analysis (Supplementary Fig. S1, Supplementary Table S4) were indistinguishable from the original result that included Hurricane Isaac (compare to Fig. 4, Supplementary Table S3). Rangoonwala et al.^13^ came to the same conclusion in their independent analysis, indicating that Hurricane Isaac had little erosion influence in our specific study area. Turner et al.^12^ also found that Hurricane Isaac had little influence on salt marsh shoreline retreat in the region. This is not to say that oiling impacts and hurricane influences cannot interact to increase marsh erosion; they certainly could, but it does not appear this was the case for our study sites and Hurricane Isaac.

## Wave exposure modeling, before–after-control-impact continued

**Figure 5:**
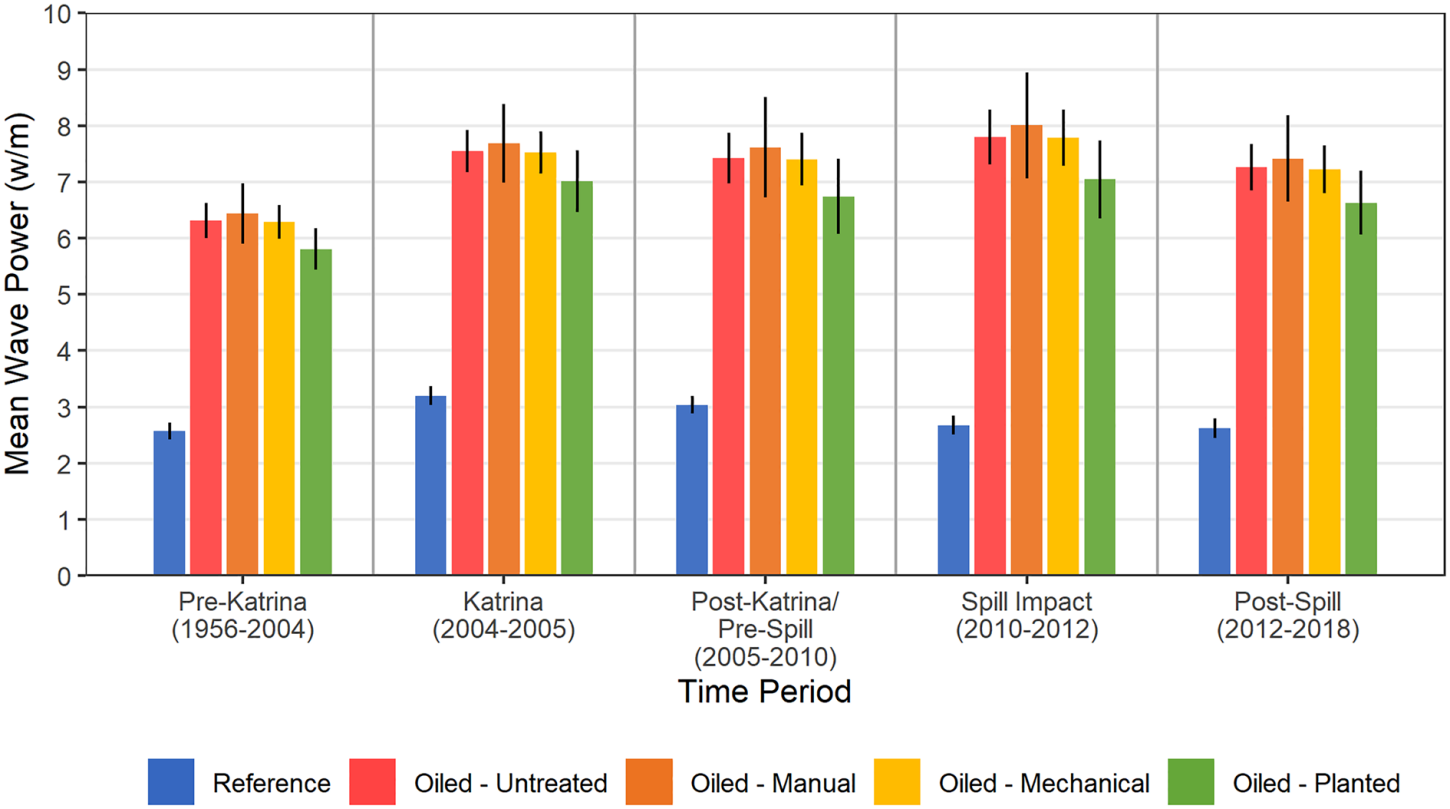
Modeled mean wave power in oiled marsh versus reference sites 1956–2018 (W m^−1^). Data are means with 90% confidence intervals, n = 5 for Reference, 9 for Oiled-Untreated, 5 for Oiled-Manual, 9 for Oiled-Mechanical, and 5 for Oiled-Mechanical-Planted treatments. Mean wave power differed among oiling/treatment categories (F_4,28_ = 52.166 *p* = 0.000); among time-periods (F_1.13,31.71_ = 396.386, *p* = 0.000); and for the interaction of oiling/treatment and time-period (F_4.53,31.71_ = 15.503, *p* = 0.000). Within each time-period mean wave power differences were observed between Reference versus each oiled class (*p* = 0.000) but were not observed among any of the oiled categories (*p* = 0.153–1.000). Between the post-Katrina/pre-spill (2005–2010) and spill impact (2010–2012) time-periods, erosion differences were observed within each of the oiled/treatment classes including reference (*p* = 0.000 to 0.004). See Supplementary Table S5 for detailed two-way mixed ANOVA results. Tukey’s test was used for all pairwise comparisons after ANOVA.

Differential wave exposure among oiled and unoiled or reference sites was the other major confounding factor raised by both Deis et al.^18^ and Challenger et al.^19^ for most *Deepwater Horizon* marsh erosion studies, including our prior work^7^. We examined this question for our study sites and time-periods by modeling wave power after Allison et al.^29^. We computed wave power for numerous scenarios of wind direction and speed based upon modeled significant wave height (Hs) and then calculated weighted mean wave power values over the different time-periods. We found that mean wave power (watts [W m^−1^]) did differ for our reference versus oiled sites, similarly across all time-periods, whereas the oiled sites with various treatments (including planting) did not differ from each other within any time-periods (Fig. 5, Supplementary Table S5). It should be recognized that mean wave power values were relatively low in both the reference and oiled sites (3 and 7 W m^−1^ on average, respectively), and the differences in wave power were small. For instance, the range of mean wave power values for coastal marshes across Louisiana is 0 to 100 W m^−1^, with the bulk of values in the 0 to 40 W m^−1^ range^29^. We would expect a 4 W m^−1^ difference in wave power to result in minor differences in marsh edge erosion. We also found differences in mean wave power among our study time-periods, however, these changes were even smaller, 0.3–0.4 W m^−1^ by oiling/treatment class for the post-Katrina/pre-spill period (2005–2010) versus the spill impact period (2010–2012). We also similarly modeled maximum wave power (e.g., wave power associated with large storms, including hurricanes, occurring over very short time-periods), which indicated much higher wave power values, but with similar relationships and even smaller differences between the reference and oiled sites. Despite the relatively consistent differences in wave power among the reference and oiled sites across time-periods, the only time-period where large differences in erosion were observed overall, was the spill impact period (2010–2012). Moreover, our Before–After-Control-Impact study design specifically controls for these types of consistent differences among impact and reference (control) sites.

For readers wary of our Before–After-Control-Impact approach who may still be concerned with differences in wave exposure between our reference and oiled sites, we offer our Before–After findings as an alternative (Fig. 4, Supplementary Table S3, see the time-period contrasts for each of the oiled categories), which can be viewed without considering our reference sites (Deis et al.^18^ took a similar Before–After approach in the interpretation of their data). A Before–After view of our data does not change our findings. Furthermore, as oiled marsh vegetation impacts are the likely primary cause of increased erosion effects from the spill, our planted sites offer another type of “reference” or “control” where wave exposure differences were not a factor. Our vegetation planting results offer powerful evidence that increased erosion was caused by the spill, that vegetation impact was a major mechanism for increased erosion, and that relatively simple and low-cost restoration planting methods can be used to substantially decrease marsh erosion following oil spills.

## Further discussion and conclusions

Several benefits of our tightly controlled study, such as: (a) well-documented continuous and consistent heavy oiling across all our oiled study sites; (b) known standardized cleanup and planting treatments intermixed across the oiled study area; (c) oiled and reference sites located as near to one another as possible along the same marsh shoreline; and (4) closely coupled field and remote sensing measurements, also relate to the main drawback of our work—our study area was small and localized and the results may not be widely transferable to larger areas (although ~ 73–109 km of marsh shorelines had oiling conditions similar to our study area and ~ 27 km of those had intensive marsh cleanup^1,23,30^). We think a main value of our field experiment and Before–After-Control-Impact approach is that they contribute important fine-scale reinforcement to the various similar wider-scale studies, such as Gibeaut et al.^8^, Silliman et al.^14^, and Beland et al^16^. We considered the next step of expanding our study across a wider geography; however, for the examination of *Deepwater Horizon* oil spill effects on marsh erosion (but not cleanup treatment or planting influences), we feel this has largely been done by others, particularly in one specific case^16^. The Beland et al.^16^ remote sensing study, spanning northern Barataria Bay salt marshes, looked at a multiyear post-Katrina/pre-spill period (2006–2010), a spill impact period (2010–2013), and a post spill period (2013–2016), including a range of oiling levels from unoiled to heavily oiled marshes, and arrived at similar erosion rates and conclusions to our present work. The Beland et al.^16^ study was largely dismissed in one recent review^18^ along with other remote sensing studies, due to concerns about the accuracy of their shoreline mapping methods, particularly the possible inability to identify the seaward edge of marsh shorelines that were oiled and denuded of vegetation. However, in our review of Beland et al.^16^, including specific discussions of their shoreline extraction and ground-truthing methods (Michael Beland, pers. comm.), we are confident they were able to accurately reflect shoreline position, including for oiled and unvegetated marsh platforms (e.g., they used finer-scale imagery than many other studies, they were aware of and able to accurately classify unvegetated marsh shorelines, and they conducted appropriate field-based data validation). Our present work, in conjunction with Beland et al.^16^, provides compelling evidence that the *Deepwater Horizon* oil spill resulted in substantially increased marsh erosion over 2 to 3 years in heavily oiled marshes. In addition, our current work confirms differences in erosion among oiled marsh cleanup treatment methods, with lesser erosion observed for manual versus mechanical treatments. Importantly, our study directly shows that shoreline stabilization or emergency restoration by vegetation planting can be highly effective in reducing oiled marsh shoreline erosion, and that positive influences of planting can extend beyond the immediate spill impacts. Logical next steps may include using remote sensing Before–After-Control-Impact approaches to examine marsh erosion in other locations affected by the *Deepwater Horizon* oil spill, as well as examining wider-scale influences of oil spill cleanup treatments and post-spill restoration on marsh erosion.

## Methods

Brief methods are included above under each component of the study coupled with corresponding results and discussion. Statistical analyses for erosion rates and wave power used two-way mixed ANOVAs with oiling/treatment class as the between-subjects factor and time-period (year or groups of years) as the within-subjects factor. ANOVA pairwise comparisons were made using Tukey’s test. Welch’s two sample t-test was used for the comparison of soil shear strength. We considered statistical significance as *p* ≤ 0.10. Descriptive statistics, ANOVA tables and t-tests, and post-ANOVA pairwise test results are reported in full in Supplementary Tables S1–S5. See the Supplementary Methods for background and details on the study area, data collection, data sources, and analysis. Authorizations and permits to conduct the marsh cleanup treatments, restoration planting, and subsequent monitoring and research were provided by and through the *Deepwater Horizon* Unified Command and Emergency Response (U.S. Coast Guard, the State of Louisiana, and BP). The study area is privately owned; access permits were obtained from the landowner prior to conducting all work.

## Supplementary Information

Supplementary Information 1.

Supplementary Information 2.

Supplementary Information 3.

Supplementary Information 4.

Supplementary Information 5.

Supplementary Information 6.

Supplementary Information 7.

## Data Availability

Data are publicly available through the GoMRI Information & Data Cooperative (GRIIDC) at https://data.gulfresearchinitiative.org/ (https://doi.org/10.7266/VF524143).

## References

[1] Nixon Z (2016). Shoreline oiling from the *Deepwater Horizon* oil spill. Mar. Pollut. Bull..

[2] Mendelssohn IA (2012). Oil impacts on coastal wetlands: Implications for the Mississippi River Delta ecosystem after the *Deepwater Horizon* oil spill. Bioscience.

[3] Editorial. Valuing wetlands. *Nat. Geosci.* **14**, 111 (2021).

[4] Couvillion, B. R., Beck, H., Schoolmaster, D. & Fischer, M. Land area change in coastal Louisiana 1932 to 2016. US Geological Survey Scientific Investigations Map 3381, https://pubs.er.usgs.gov/publication/sim3381 (2017).

[5] Silliman BR (2012). Degradation and resilience in Louisiana salt marshes after the BP-Deepwater Horizon oil spill. Proc. Natl. Acad. Sci. U. S. A..

[6] McClenachan G, Turner RE, Tweel AW (2013). Effects of oil on the rate and trajectory of Louisiana marsh shoreline erosion. Environ. Res. Lett..

[7] Zengel S (2015). Heavily oiled salt marsh following the *Deepwater Horizon* oil spill, ecological comparisons of shoreline cleanup treatments and recovery. PLoS ONE.

[8] Gibeaut, J. C., Nixon, Z. & Rouhani, S. Shoreline change analysis of oiled and treated shorelines in upper Barataria Bay. *Deepwater Horizon* Programmatic Damage Assessment—Programmatic Environmental Impact Statement Administrative Record, Technical Report DWH-AR0270436, https://www.fws.gov/doiddata/dwh-ar-documents/901/DWH-AR0270436.pdf (2015).

[9] Lin Q (2016). Response of salt marshes to oiling from the *Deepwater Horizon* spill: Implications for plant growth, soil surface-erosion, and shoreline stability. Sci. Total Environ..

[10] Beland M (2016). Mapping changing distributions of dominant species in oil-contaminated salt marshes of Louisiana using imaging spectroscopy. Remote Sens. Environ..

[11] McClenachan, G. Coastal ecosystem resiliency after major disturbances. Dissertation, Louisiana State University, https://digitalcommons.lsu.edu/gradschool_dissertations/3161/ (2016).

[12] Turner RE, McClenachan G, Tweel AW (2016). Islands in the oil: Quantifying salt marsh shoreline erosion after the *Deepwater Horizon* oiling. Mar. Pollut. Bull..

[13] Rangoonwala A, Jones CE, Ramsey E (2016). Wetland shoreline recession in the Mississippi River Delta from petroleum oiling and cyclonic storms. Geophys. Res. Lett..

[14] Silliman BR (2016). Thresholds in marsh resilience to the *Deepwater Horizon* oil spill. Sci. Rep..

[15] Khanna S (2017). Marsh loss due to cumulative impacts of Hurricane Isaac and the *Deepwater Horizon* oil spill in Louisiana. Remote Sens..

[16] Beland M (2017). Oiling accelerates loss of salt marshes, southeastern Louisiana. PLoS ONE.

[17] Powers SP (2017). Ecosystem services are lost when facilitation between two ecosystem engineers is compromised by oil. Mar. Ecol. Prog. Ser..

[18] Deis DR, Mendelssohn IA, Fleeger JW, Bourgoin SM, Lin Q (2019). Legacy effects of Hurricane Katrina influenced marsh shoreline erosion following the *Deepwater Horizon* oil spill. Sci. Total Environ..

[19] Challenger GE, Gmur S, Taylor E (2021). A review of Gulf of Mexico coastal marsh erosion studies following the 2010 *Deepwater Horizon* oil spill and comparison to over 4 years of shoreline loss data from Fall 2010 to Summer 2015. Mar. Pollut. Bull..

[20] Bernik BM, Lumibao CY, Zengel S, Pardue J, Blum MJ (2021). Intraspecific variation in landform engineering across a restored salt marsh shoreline. Evol. Appl..

[21] Smith EP (2002). BACI design. Encycl. Environmetr..

[22] Smokorowski KE, Randall RG (2017). Cautions on using the before–after-control-impact design in environmental effects monitoring programs. Facets.

[23] Zengel S (2021). Planting after shoreline cleanup treatment improves salt marsh vegetation recovery following the *Deepwater Horizon* oil spill. Ecol. Eng..

[24] Zengel S (2022). Meta-analysis of salt marsh vegetation impacts and recovery: A synthesis following the *Deepwater Horizon* oil spill. Ecol. Appl..

[25] Sasser CE (2018). Relationships of marsh soil strength to belowground vegetation biomass in Louisiana coastal marshes. Wetlands.

[26] Silliman BR (2019). Field experiments and meta-analysis reveal wetland vegetation as a crucial element in the coastal protection paradigm. Curr. Biol..

[27] Valentine K, Mariotti G (2019). Wind-driven water level fluctuations drive marsh edge erosion variability in microtidal coastal bays. Cont. Shelf Res..

[28] Himmelstoss, E. A., Henderson, R. E., Kratzmann, M. G. & Farris, A. S. Digital shoreline analysis system (DSAS) version 5.0 user guide. US Geological Survey Open-File Report 2018–1179, https://pubs.er.usgs.gov/publication/ofr20181179 (2018).

[29] Allison, M. *et al.* Coastal master plan, model improvement plan, attachment C3–2, marsh edge erosion. Louisiana Coastal Protection and Restoration Authority, http://coastal.la.gov/wp-content/uploads/2017/04/Attachment-C3-2_FINAL_02.23.2017.pdf (2017).

[30] Goovaerts P, Wobus C, Jones R, Rissing M (2016). Geospatial estimation of the impact of *Deepwater Horizon* oil spill on plant oiling along the Louisiana shorelines. J. Environ. Manag..

