# Supplementary material for: Accelerated marsh erosion following the Deepwater Horizon oil spill confirmed, ameliorated by planting
Source: Sci Rep. 2022 Aug 13;12:13802. doi: 10.1038/s41598-022-18102-1 (PMC9376092; doi:10.1038/s41598-022-18102-1)
Supplement: Supplementary file 1 — Supplementary Information 1. [file 41598_2022_18102_MOESM1_ESM.pdf]

Accelerated Marsh Erosion Following the *Deepwater Horizon* Oil Spill Confirmed, Ameliorated by Planting

Scott Zengel, Zachary Nixon, Jennifer Weaver, Nicolle Rutherford, Brittany M. Bernik, Jacqueline Michel

Supplementary Figure

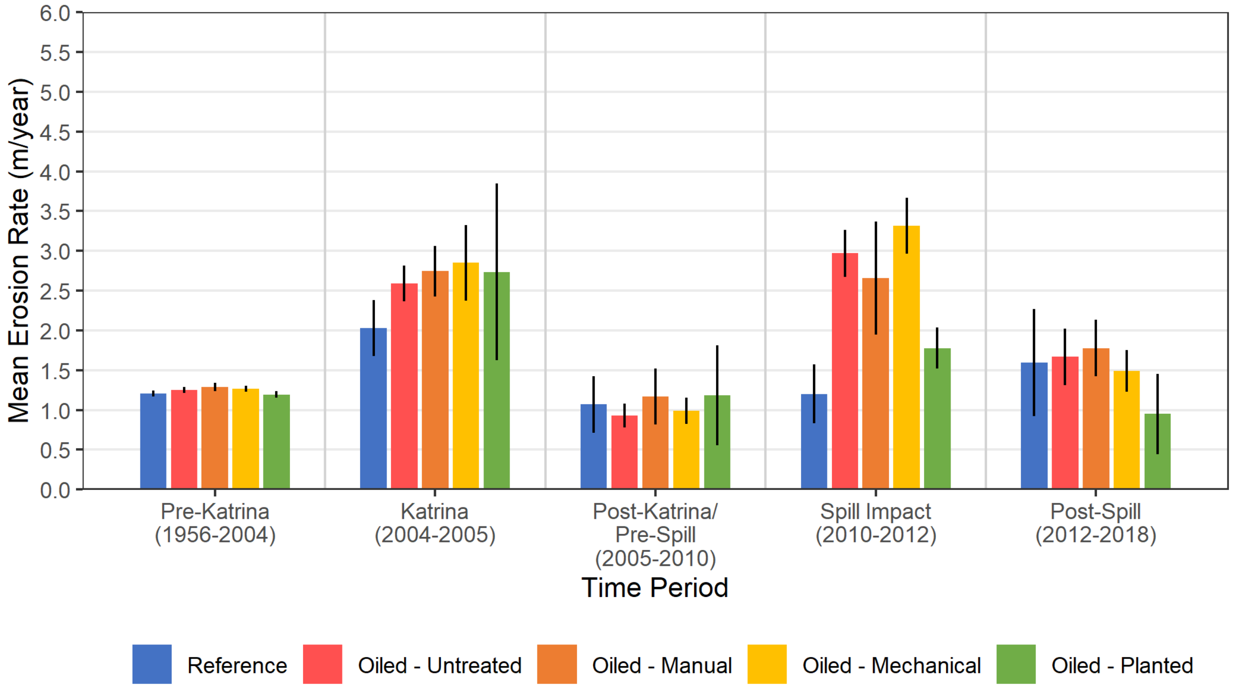

Supplementary Figure S1. Remote sensing measured marsh shoreline erosion rates 1956-2018 (m yr^{-1}), without the influence of Hurricane Isaac in 2012. Data are means with 90% confidence intervals, n= 5 for Reference, 9 for Oiled-Untreated, 5 for Oiled-Manual, 9 for Oiled-Mechanical, and 5 for Oiled-Mechanical-Planted treatments. Marsh erosion rates differed among oiling/treatment categories ($F_{4,28}$ = 9.207, p = 0.000); among time-periods ($F_{2.42,67.77}$ = 62.222, p = 0.000); and for the interaction of oiling/treatment and time-period ($F_{9.68,67.77}$ = 4.518, p = 0.000). In the post-Katrina/pre-spill period (2005-2010) erosion differences were not observed among any oiling/treatment classes, including Reference (p = 0.873 to 1.000). In the spill impact period (2010-2012) erosion differences were observed between: Reference versus all oiled classes (p = 0.000) except Planted (p = 0.324); and between Planted versus all other oiled classes (p = 0.000 to 0.048). In the post-spill period (2012-2018) erosion differences were observed between: Planted versus Manual treatment and Untreated (p = 0.070 and 0.077). Between the post-Katrina/pre-spill and spill impact time-periods, erosion differences were not observed within the Reference (p = 0.992) or Planted (p = 0.276) classes; however, differences were observed between these time-periods within each of the other oiled classes (p = 0.000 in all cases). See Supplementary Table S4 for detailed two-way mixed ANOVA results. Tukey's test was used for all pairwise comparisons after ANOVA.
