# Supplementary material for: Accelerated marsh erosion following the Deepwater Horizon oil spill confirmed, ameliorated by planting
Source: Sci Rep. 2022 Aug 13;12:13802. doi: 10.1038/s41598-022-18102-1 (PMC9376092; doi:10.1038/s41598-022-18102-1)
Supplement: Supplementary file 2 — Supplementary Information 2. [file 41598_2022_18102_MOESM2_ESM.pdf]

Accelerated Marsh Erosion Following the *Deepwater Horizon* Oil Spill Confirmed, Ameliorated by Planting

Scott Zengel, Zachary Nixon, Jennifer Weaver, Nicolle Rutherford, Brittany M. Bernik, Jacqueline Michel

Supplementary Methods

Study Area and Oiling/Treatment Classes

The study was conducted in the "marsh treatment test area" established under the *Deepwater Horizon* oil spill emergency response, located in northern Barataria Bay, Louisiana, USA. Salt marshes in this area are naturally dominated by *Spartina alterniflora* (smooth cordgrass) and to a lesser degree *Juncus roemerianus* (black needlerush). Initial heavy oiling of salt marshes in the study area began in early June 2010. Following oil source control and the end of on-water oil collection, field assessments in Fall 2010 indicated a continuous 6-13 meters (m) wide oiling band along the marsh shoreline in the study area, with heavily oiled wrack and vegetation mats overlying a 2-3 centimeter (cm) layer of emulsified oil on the marsh surface with ~90-100% oil cover[1,2]. Total polycyclic aromatic hydrocarbons (tPAH) averaged 833 milligrams per kilogram (mg kg^{-1}) in the surface oil layer and 260 mg kg^{-1} in the underlying marsh soils in 2011[1,3]. Our oiled study sites were located within this continuous and consistent band of heavy oiling. For information on changes in oiling and vegetation conditions over time see Zengel et al.[1,2].

Manual oiled marsh cleanup treatment involved raking, cutting, and removal of oiled wrack, oiled vegetation mats, and underlying oil on the substrate by small crews using hand tools[1,2,3]. Hand crews used walking boards to minimize foot traffic on the marsh surface. Mechanical cleanup treatment involved mechanized grappling to remove oiled wrack and mechanized raking, cutting, and scraping to remove or reduce oiled vegetation mats and oil on the substrate[1,2,3]. The mechanical treatments were applied using long-reach hydraulic arms mounted on shallow-draft barges and large airboats stationed just seaward of the marsh shoreline. Marsh cleanup treatments were not applied in the untreated and

reference sites. Vegetation planting in a subset of mechanically treated sites involved hand-planting individual bare root *Spartina alterniflora* stems at a density of ~2-3 stems m^{-2} following mechanical cleanup treatment[2,4,5]. Planted sites were largely devoid of live vegetation before planting, due to oil impacts and cleanup treatments. Planting material consisted of a transplanted local wild *Spartina alterniflora* variety from Bay Jimmy which was native to the study area. No fertilizer was applied.

The oiled manual treatment sites (5 replicates) and oiled untreated sites (9 replicates) were randomly established within the continuous heavy oiling band during October to December 2010. The manual treatments were applied in December 2010. The untreated sites were left untreated for the duration of the study. The oiled mechanical treatment sites (9 replicates) were randomly established in areas that received operational scale mechanical treatments in May-June 2011 under the emergency response. The reference sites (5 replicates) were randomly located along the nearest contiguous and comparable section of shoreline in the study area. The reference sites had minor to no oiling during the study period and intact vegetation structure. The oiled, mechanically treated, and planted sites (5 replicates) were randomly located in mechanically treated areas in Summer and early Fall 2011.

Marsh Erosion Field Methods

Field erosion measurements for this study were conducted in September/early October over seven years, from 2010-2016, representing a time interval of six years following initial oiling and five years following mechanical treatments. Erosion was measured during each field sampling event by locating the position of the shoreline, considered to be the marsh scarp or location of steepest vertical slope in elevation, using tape measures and a high-accuracy, differentially-corrected Global Positioning System (GPS) unit with a horizontal error of +/- 10 cm. Shoreline position was collected along two transects per site. An annual erosion rate was then computed for each study site by measuring the distance between shoreline positions along each of two transects, normalizing for the time interval, and averaging the resulting rate for both transects.

Shear Strength Field Methods

Lin et al. measured soil shear strength during the 2011-2012 period within a subset of our study sites using a 3.3-cm diameter shear vane attached to a direct reading torque gauge. Soil shear strength was measured at a soil depth of 6 cm at two locations within each site. See Lin et al.[6] for additional details.

Remote Sensing Erosion Analysis Methods

We reviewed all digital aerial imagery available via the USGS Earth Explorer web portal[7] for inclusion in this analysis, including imagery from the National High-Altitude Photography (NHAP) Program, the National Aerial Photography Program (NAPP), the National Agriculture Imagery Program (NAIP), and the MC-252 Natural Resources Damage Assessment (NRDA) aerial imagery archives. We also obtained selected high-resolution images from Google Earth for some years where no aerial imagery was publicly available. Some available imagery was not used for analysis where higher resolution imagery from the same year was available. Supplementary Methods Table 1 summarizes the dates, sources, and characteristics of the aerial imagery selected for analysis.

To ensure maximum precision in erosion measurements, we corrected[8] for co-registration error in aerial imagery mosaics. All imagery was re-projected to a single coordinate system and georeferenced using a minimum of 10 points within the analysis area and a forward projective transformation. All imagery was co-registered to the NRDA imagery acquired on 10/7/2010. Because human-made or permanently constructed features are not present in the analysis area, tidal creek intersections were used as ground control points for the purpose of georeferencing and rectification[9,10]. Average Root Mean Square Error (RMSE) was approximately 0.47 m.

After correcting for co-registration error, all imagery was processed to extract linear shoreline vectors. For imagery with a near-infrared (NIR) band, a binary threshold of the NIR band was computed using Otsu's method[11]. For imagery without a near infrared band, a composite brightness was computed

Supplementary Methods Table 1. Details of aerial imagery used in the study. For analysis of impact excluding Hurricane Isaac, the 9/1/2012 imagery and shoreline was excluded from the spill impact period.

Date Acquired	Source	Resolution (m)	Spectral Bands	Registration RMS Error (m)	Total Positional Uncertainty (m)	Time Period
2/22/1956	NHAP	0.8	1	1.86	2.02	
5/5/1972	NHAP	3	3 (CIR)	1.61	3.40	
10/27/1983	NHAP	4	3 (CIR)	1.12	4.15	
10/26/1989	NAPP	3	3 (CIR)	1.80	3.50	Pre-Katrina
1/30/1994	NAPP	2.8	1	1.40	3.13	
1/24/1998	NAPP	1	4	0.49	1.12	
1/21/2004	NAPP	1	3 (CIR)	0.26	1.03	
11/2/2005	NAPP	1	3 (CIR)	0.21	1.02	Katrina
5/20/2006	Google Earth	0.5	3	0.29	0.58	
7/22/2007	NAIP	1	3	0.28	1.04	Post-Katrina/ Pre-Spill
10/1/2008	USGS-LA	1	4	0.28	1.04	
8/15/2009	NAIP	1	3	0.18	1.02	
5/25/2010	NRDA	0.35	4	0.21	0.41	
10/7/2010	NRDA	0.33	4	Reference	0.33	
5/6/2011	NRDA	0.33	4	0.13	0.35	
10/1/2011	NRDA	0.33	4	0.22	0.40	Spill Impact
5/10/2012	NRDA	0.33	4	0.17	0.37	
8/24/2012	NRDA	0.33	4	0.12	0.35	
9/1/2012	NRDA	0.33	4	0.26	0.42	
5/14/2013	NRDA	0.33	4	0.11	0.35	
10/7/2013	NAIP	1	4	0.39	1.07	
5/3/2015	NAIP	1	4	0.34	1.06	Post-Spill
11/202016	Google Earth	0.33	3	0.20	0.39	
9/8/2017	NAIP	1	4	0.26	1.03	
8/12/2018	Google Earth	0.33	3	0.27	0.42	

for all bands and a threshold was computed in a similar fashion. The resulting binary rasters were cleaned with a focal majority filter using 8 neighbors, and then a raster boundary cleaning algorithm, and then converted to vector polygons with smoothing using ESRI ArcGIS 10.8. The resulting algorithmically derived shorelines were then manually edited to ensure accurate shoreline features

were being extracted, particularly in imagery acquired immediately after oil stranding and vegetation impacts at the seaward marsh edge.

We measured changes in the position of the extracted marsh shorelines, and computed shoreline rate-of-change statistics, using the Digital Shoreline Analysis System (DSAS) version 5.0[12]. This software has been used to analyze changes in shoreline position in a range of coastal systems, including estuarine and lagoon boundaries[13] and coastal wetlands[10]. Uncertainty for each derived shoreline was estimated after the methods described by Ruggiero et al.[14] for aerial imagery-derived shorelines, where georeferencing uncertainty is taken as the RMSE registration error in m, and digitization error is taken as the pixel size of the digital image in m (Supplementary Methods Table 1). We defined the shoreline as the vertical or near-vertical scarp at the seaward edge of the vegetated area, or the same scarp at the margin of recently denuded marsh platform. Because the tidal range is microtidal and the vegetation canopy is nearly always present above any water on the marsh platform, we discount potential tidal datum bias and water level-related sources of uncertainty. Images acquired during and after the spill, where denuded marsh platform was present, were carefully compared to the field GPS data to ensure the shorelines extracted from imagery for these years accurately reflected the position of the scarp at the edge of the marsh platform.

We established an onshore baseline by buffering all shorelines at 30 m and simplifying resulting buffer extent[15,16,] removing 90% of the vertices, and smoothed by computing Bezier curves between vertices. Transects were cast in DSAS from the smoothed onshore baseline every 1 m using an azimuth smoothing range of 50 m. Transects were assigned to individual sampling sites by spatial intersection. Transects that intersected more than one site were removed from further analysis. Shorelines were grouped together into time periods and change rates in each time-period were determined using DSAS. Shoreline change rates for time periods with more than two shorelines were estimated using the weighted linear regression rate method, where the rate is computed as the slope of a least-squares

regression line fit to all shoreline positions along a transect, with each position weighted by the positional uncertainty of that shoreline. Shoreline change rates for time periods with only two shorelines were estimated using the end point rate method, where the rate is computed as distance between shoreline positions along a transect divided by the time elapsed between the two shorelines. Shoreline change rates were averaged over all transects for each site prior to further analysis.

Wave Power Analysis

Wave power is directly related to erosion and morphology of wetland shorelines both globally and in the region[17,18,19,20,21,22,23]. In northern Barataria Bay, nearly all wave power impinging on shorelines is likely a result of locally generated wind-waves rather than oceanic swell (see Johnson[24] and Everett et al.[25] for investigations in comparable areas in Terrebonne Bay). Further, in Barataria Bay and other enclosed marsh basins, the majority of marsh edge erosion over annual or longer time-scales is caused by wind-waves generated by relatively common wind conditions rather than tropical storms or other relatively rare high energy events[22,26,27]. To estimate wave power at a transect representing each plot location over each time period of interest, we used a probabilistic method for conducting steady-state empirical shallow-water wave modeling wherein 240 different climatological scenarios representing different wind speeds and directions with different occurrence frequencies were used to parameterize empirical shallow-water wave models.

Fetch was computed separately for each time-period of interest. Land and water data were constructed for each time-period by reclassifying the land-loss data were generated by Couvillion et al.[28] to create different 30 m land-water grids at the following time steps: 1956, 2004, 2006, 2010, 2013, and 2016. At each site, a single transect was generated, running through the centroid of the site, and having as its azimuth the average of all the DSAS transects that also intersected that site. For each site centroid transect, a single location was generated at 50 m from the shoreline position for each of the following years that approximately correspond to the time steps used for the land-water grids: 1956, 2004, 2005,

2010, 2012, and 2018. A location 50 m offshore the shoreline corresponding to each time step was used to compute fetch representing a location approximately far enough from the marsh scarp to realistically represent incident wind waves[22] and where water depth is asymptotically approaching equilibrium depth[29]. At each transect offshore location, fetch was computed in 15° increments using the effective fetch methodology[30] with 9 cosine-weighted radials in 3 increments about the primary angle. Fetch was computed using the Waver package[31] for the R statistical computing language. A maximum fetch of 30 kilometers (km) was assumed in any direction. Fetch for each time-period was then computed as the average of fetch calculated at each bracketing time step.

Mariotti et al.[32] noted that wind statistics derived from stations on or adjacent to the shoreline often do not reflect wind conditions in open water areas of nearby bays and estuaries, and specifically recommended the use of wind data from the "BURL1 station" of the NOAA National Data Buoy Center (NDBC) for analyses of wind climate in the interior portions of Barataria Bay. As such, we compiled all available (1986 to 2021) hourly wind data from the BURL1 station and converted those to standard wind speeds (m s^{-1}) at 10 m using the correction described in Mariotti et al.[32]. For each time-period, we then computed wind summary statistics within 24 directional bins at 15° increments, and 10 velocity bins with breakpoints at 0, 2, 4, 6, 8, 10, 15, 20, 25, 30, and 35 m s^{-1}, yielding a total of 240 individual wind speed and direction scenarios.

Wave Power Model

For each transect offshore location, time-period, fetch direction, and wind speed and direction scenario we computed wave power statistics, generally following the methods outlined in Allison et al.[21], which were similar to methods used by many other researchers who have investigated the relationship between wave power and marsh erosion[10,18,19,20,22,23]. Following this approach, we computed wave energy and period using the empirical equations of Young and Verhagen[33] for a given wind speed and fetch, using an assumed water depth of 0.8 m[22]. We then computed wave energy flux or power in units

of watts per m (W m^{-1}), for each transect offshore location and evaluated modeling scenario. This calculation followed the shallow-water approximations from Airy linear wave theory using model-predicted energy, significant wave height and peak wave period after Holthuijsen[34] as:

$$P = E \; C_g \, \rho g \cos \theta$$

where *P* is power or wave energy flux per unit of wave-crest length or power in units of W m^{-1}, ρ is the density of seawater (1027 kg m^{-3}) and g the acceleration of gravity (9.8 m s^{-2}), C_g is the wave group velocity in m s^{-1}, and θ is the difference between the angle of wave approach and the angle normal to the shoreline. Winds blowing from greater than 90° from the shore-normal direction (from over land) were not considered to generate impinging wind waves. We then computed the maximum and weighted mean significant wave height (Hs) and wave power for each transect offshore location within each time-period. Weighted means were computed for each transect offshore location by summing the products of each scenario wave power and the scenario frequency, over all scenarios, for that time-period.

Statistical Analysis

Erosion rates and wave power were plotted by oiling/treatment class per time-period (years or groups of years) as means ± 1 standard error (SE). Two-way mixed ANOVAs were used for statistical analyses with oiling/treatment class as the between-subjects factor and time-period as the within-subjects factor. Greenhouse-Geiser corrections were applied in cases where the sphericity assumption was violated according to Mauchly's test. Post-hoc pairwise comparisons were made using Tukey's test when the ANOVA results indicated potential differences. Welch's two sample t-test was used to compare soil shear strength measured at reference and oiled sites. All tests were two-tailed. We generally considered statistical significance as $p \leq 0.10$; however, based on recent guidance[35,36], we did not use these values as strict cutoff points, choosing instead to form our overall interpretations based on the combination of the plotted data, including visual trends and tendencies in the data, and the

statistical results. Analyses were conducted in R version 3.6.3. ANOVA and t-test statistics, degrees of freedom, p-values, and summaries for pairwise comparisons are reported in the figure captions with the corresponding data figures. Descriptive statistics, ANOVA and t-test tables, and post-ANOVA pairwise test results are reported in full in Supplementary Tables S1-S5.

References

1. Zengel, S. et al. Heavily oiled salt marsh following the *Deepwater Horizon* oil spill, ecological comparisons of shoreline cleanup treatments and recovery. *PLOS One* **10**(7), e0132324 (2015).
2. Zengel, S. et al. Planting after shoreline cleanup treatment improves salt marsh vegetation recovery following the *Deepwater Horizon* oil spill. Ecol Eng **169**, 106288 (2021).
3. Zengel, S. A. & Michel, J. *Deepwater Horizon* oil spill: Salt marsh oiling conditions, treatment testing, and treatment history in northern Barataria Bay, Louisiana. NOAA Technical Memorandum NOS OR&R 42, https://repository.library.noaa.gov/view/noaa/380 (2013).
4. Bernik, B. M. Ecosystem consequences of genetic variation in the salt marsh grass *Spartina alterniflora*. PhD Dissertation, Tulane University (2015).
5. Bernik, B. M. et al. Intraspecific variation in landform engineering across a restored salt marsh shoreline. *Evol Appl* **14**(3), 685-697 (2021).
6. Lin, Q. et al. Response of salt marshes to oiling from the Deepwater Horizon spill: Implications for plant growth, soil surface-erosion, and shoreline stability. *Sci Total Environ* **557-558**, 369-377 (2016).
7. US Geological Survey. Earth Explorer. US Geological Survey Fact Sheet 083-00, https://pubs.usgs.gov/fs/2000/0083/ (2000).
8. Gibeaut, J. C., Nixon, Z. & Rouhani, S. Shoreline change analysis of oiled and treated shorelines in Upper Barataria Bay. *Deepwater Horizon* Programmatic Damage Assessment - Programmatic Environmental Impact Statement Administrative Record, Technical Report DWH-AR0270436, https://www.fws.gov/doiddata/dwh-ar-documents/901/DWH-AR0270436.pdf (2015).

9. Higinbotham, C. B., Alber, M. & Chalmers, A. G. Analysis of tidal marsh vegetation patterns in two Georgia estuaries using aerial photography and GIS. *Estuaries* **27**(4), 670-683 (2004).

10. McLoughlin, S. M., Wiberg, P. L., Safak, I. & McGlathery, K. J. Rates and forcing of marsh edge erosion in a shallow coastal bay. *Estuaries and Coasts* **38**(2), 620-638 (2015).

11. Otsu, N. A threshold selection method from gray-level histograms. *IEEE Transactions on Systems, Man, and Cybernetics* **9**(1), 62-66 (1979).

12. Himmelstoss, E. A., Henderson, R. E., Kratzmann, M. G. & Farris, A. S. Digital shoreline analysis system (DSAS) version 5.0 user guide. US Geological Survey Open-File Report 2018–1179, https://pubs.er.usgs.gov/publication/ofr20181179 (2018).

13. Cowart, L., Walsh, J. & Corbett, D. R. Analyzing estuarine shoreline change: A case study of Cedar Island, North Carolina. *Journal of Coastal Research* **26**(5), 817-830 (2010).

14. Ruggiero, P. et al. National assessment of shoreline change: historical shoreline change along the Pacific Northwest coast. US Geological Survey (2013).

15. Dunn, A., Hanson, B. A. & Seeger, C. J. Simplifying and editing vector data with mapshaper.org. *Extension and Outreach Publications 192*, Iowa State University (2016).

16. Visvalingam, M. & Whyatt, J. Line generalisation by repeated elimination of the smallest area. Discussion paper: Cartographic Information Systems Research Group, The University of Hull (1992).

17. Schwimmer, R. A. Rates and processes of marsh shoreline erosion in Rehoboth Bay, Delaware, USA. *Journal of Coastal Research* ***17**(3)*, 672-683 (2001).

18. Mariotti, G. et al. Influence of storm surges and sea level on shallow tidal basin erosive processes. *Journal of Geophysical Research: Oceans* **115**, C11012 (2010).

19. Marani, M., D'Alpaos, A., Lanzoni, S. & Santalucia, M. Understanding and predicting wave erosion of marsh edges. *Geophysical Research Letters* **38**, L21401 (2011).

20. Trosclair, K. J. Wave transformation at a saltmarsh edge and resulting marsh edge erosion: observations and modeling. Master's Thesis, University of New Orleans (2013).
21. Allison, M. et al. Coastal master plan, model improvement plan, attachment C3–2, marsh edge erosion. Louisiana Coastal Protection and Restoration Authority, http://coastal.la.gov/wp-content/uploads/2017/04/Attachment-C3-2_FINAL_02.23.2017.pdf (2017).
22. Valentine, K. & Mariotti, G. Wind-driven water level fluctuations drive marsh edge erosion variability in microtidal coastal bays. *Continental Shelf Research* **176**, 76-89 (2019).
23. Finotello, A. et al. Control of wind-wave power on morphological shape of salt marsh margins. *Water Science and Engineering* **13**(1), 45-56 (2020).
24. Johnson, C. L. The influence of soil properties on marsh edge erosion. Master's Thesis, Louisiana State University (2016).
25. Everett, T., Chen, Q., Karimpour, A. & Twilley, R. Quantification of swell energy and its impact on wetlands in a deltaic estuary. *Estuaries and Coasts* **42**(1), 68-84 (2019).
26. Watzke, D. A. Short-term evolution of a marsh island system and the importance of cold front forcing, Terrebonne Bay, Louisiana. Master's Thesis, Louisiana State University (2004).
27. Leonardi, N., Ganju, N.K. & Fagherazzi, S. A linear relationship between wave power and erosion determines salt-marsh resilience to violent storms and hurricanes. *Proceedings of the National Academy of Sciences USA* **113**(1), 64-68 (2016).
28. Couvillion, B. R., Beck, H., Schoolmaster, D. & Fischer, M. Land area change in coastal Louisiana 1932 to 2016. US Geological Survey Scientific Investigations Map 3381, https://pubs.er.usgs.gov/publication/sim3381 (2017).
29. Wilson, C. A. & Allison, M. A. An equilibrium profile model for retreating marsh shorelines in southeast Louisiana. *Estuarine, Coastal and Shelf Science* **80**(4), 483-494 (2008).
30. United States Army Corps of Engineers (USACE). Coastal Engineering Manual (2002).

31. Marchand, P. & Gill, D. Waver, R package to calculate fetch and wave energy. R package version 0.2.1., https://github.com/pmarchand1/waver (2018).

32. Mariotti, G. et al. Biased wind measurements in estuarine waters. *Journal of Geophysical Research: Oceans* **123**(5), 3577-3587 (2018).

33. Young, I. R. & Verhagen, L. The growth of fetch limited waves in water of finite depth. Part 1. Total energy and peak frequency. *Coastal Engineering* **29**(1-2), 47-78 (1996).

34. Holthuijsen, L. H. *Waves in Oceanic and Coastal Waters*. Cambridge University Press (2010).

35. Wasserstein, R.L., Schirm, A.L. & Lazar, N.A. 2019. Moving to a world beyond "p< 0.05". *The American Statistician* **73**(sup1), 1-19 (2019).

36. Smith, E.P. Ending reliance on statistical significance will improve environmental inference and communication. *Estuaries and Coasts* **43**(1), 1-6 (2020).
